# Current Trends and Research Topics Regarding Intestinal Organoids: An Overview Based on Bibliometrics

**DOI:** 10.3389/fcell.2021.609452

**Published:** 2021-08-03

**Authors:** Meng-Meng Zhang, Ke-Lu Yang, Yan-Cheng Cui, Yu-Shi Zhou, Hao-Ran Zhang, Quan Wang, Ying-Jiang Ye, Shan Wang, Ke-Wei Jiang

**Affiliations:** ^1^Department of Gastroenterological Surgery, Peking University People’s Hospital, Beijing, China; ^2^Laboratory of Surgical Oncology, Beijing Key Laboratory of Colorectal Cancer Diagnosis and Treatment Research, Peking University People’s Hospital, Beijing, China; ^3^Evidence-Based Nursing Center, School of Nursing, Lanzhou University, Lanzhou, China

**Keywords:** organoid, intestinal stem cell, bibliometric analysis, overview, preclinical models

## Abstract

Currently, research on intestinal diseases is mainly based on animal models and cell lines in monolayers. However, these models have drawbacks that limit scientific advances in this field. Three-dimensional (3D) culture systems named organoids are emerging as a reliable research tool for recapitulating the human intestinal epithelium and represent a unique platform for patient-specific drug testing. Intestinal organoids (IOs) are crypt–villus structures that can be derived from adult intestinal stem cells (ISCs), embryonic stem cells (ESCs), or induced pluripotent stem cells (iPSCs) and have the potential to serve as a platform for individualized medicine and research. However, this emerging field has not been bibliometric summarized to date. Here, we performed a bibliometric analysis of the Web of Science Core Collection (WoSCC) database to evaluate 5,379 publications concerning the use of organoids; the studies were divided into four clusters associated with the current situation and future directions for the application of IOs. Based on the results of our bibliometric analysis of IO applications, we systematically summarized the latest advances and analyzed the limitations and prospects.

## Introduction

Recent progressive improvements in personalized medicine as a result of substantial developments in molecular biology and genetic research indicate the need for preclinical studies. Cell lines grown in monolayers, patient-derived tumor xenografts (PDTXs), and genetically engineered mouse models (GEMMs) are commonly used in experimental research. However, most of these models fail to phenocopy the response of diseases and drugs directly (see Table 1); human cell lines originate from a single type of cancer or embryonic cells due to strong selection bias (e.g., normal epithelium-derived cell lines are immortalized by virus transformation), which cannot recreate complex cell–cell interactions, heterogeneous environments, and gene mutations or chromosomal abnormalities (Bein et al., 2018; Fujii and Sato, 2021); the associated costs, animal ethics, and species differences between experimental animals and humans are major issues that must be resolved (Sato and Clevers, 2013; Hickman et al., 2014; Liu et al., 2016; Bein et al., 2018; Kapalczynska et al., 2018; Pereira et al., 2018; Yin et al., 2019). Thus, identifying simpler, more robust, and widely available preclinical models for basic gastrointestinal research has rapidly become a subject of interest in recent decades.

**TABLE 1:** Advantages and disadvantages of research models.

Features	*In vitro*		*In vivo*	
	Cell lines	Organoid	CDX/PDX	GEMM
Advantages	• Homogenous • Ease of passage and maintenance • Simple media • Immortalization • High-throughput drug screens • Lowest cost	• 3D structure • Parental heterogeneity • Self-renew and self-organization • Original genetic and histological profiles • High-throughput drug screens • Fast expansion	• Tumor–stroma interaction • Angiogenesis • Integrated TME • Original genetic and histological profiles	• Amenable to relevant genetic modification • Immune competent • *In situ* cancer research
Disadvantages	• Fails to reflect the physiological environment • Tissue morphology • Lacks stromal and infiltrating cells andmicroenvironment • Does not represent heterogeneity • Gene mutation or chromosomal abnormality	• Only epithelial origin • Lacks tumor–stroma interaction	• Time- and resource-consuming • Immune suppression • Complex ethical issuesComplicated operation • Fail to reproduce species-specific effect of human • Large scale experiments not possible	

To address the associated challenges, Sato et al. (2009) cultured self-organizing three-dimensional (3D) epithelial-like structures termed intestinal organoids (IOs), which are also known as “enteroids” or “mini-gut.” Upon suspension in a luminal-rich scaffold (namely, Matrigel) with a defined set of niche factors, organoids present long-term growth and expansion. The term organoid was first mentioned in a study on tumor mechanisms in 1946 (Smith and Cochrane, 1946). Subsequently, its meaning evolved to generally refer to tissues or structures that are similar to organs, and the term has been increasingly used for *in vitro* biology. IOs are derived from tissue-resident stem/progenitor cells, including embryonic stem cells (ESCs) or induced pluripotent stem cells (iPSCs), and they recapitulate many aspects of their functionality *in vitro* (Lancaster and Knoblich, 2014). Human organoid culture protocols have been established for several additional organs or tissues, such as the stomach, esophagus, colon, pancreas, breast, liver, prostate, retina, and thyroid (Barker et al., 2010; Huch et al., 2013a,b; Zhang et al., 2013; DeWard et al., 2014; Karthaus et al., 2014; Saito et al., 2018; Dorgau et al., 2019). Compared with 2D cell lines, IOs maintain all hallmarks of the original tissue in terms of architecture, cell type, and self-renewal dynamics. Moreover, organoids exhibit superior epithelial natural physiology and have stable phenotypic and genetic characteristics (Sato and Clevers, 2013; Toden et al., 2018). Overall, the development of organoids fills the gap between genetics and patient trials. In the future, they can be subcultured and cryopreserved for a long time (Sato et al., 2009; Sato and Clevers, 2013; Hickman et al., 2014; Liu et al., 2016), which represents ideal characteristics for future basic research and therapeutic development.

As a novel and bona fide research model, IOs have raised hopes for research on disease modeling, therapeutic development, host–microbe interactions, biomolecule delivery, and intestinal biology and development. At present, a significant number of academic publications related to this relevant topic are contributed from worldwide institutions and laboratories. Since these publications are scattered, a robust conclusion of the research in IOs is needed to pull the literature together in a coherent way. Here, we first performed a bibliometric analysis to form a network map using keywords from publications on IOs and provided an in-depth and comprehensive understanding of IO research involved based on a large-scale publication. This technology-based review outlines dynamic topics in biomedical and clinical research over recent decades and focused on the limitations and strengths of IOs.

## Visualization of IOs Based on Bibliometrics

### Current Developmental Trends of IO Headings

**FIGURE 1:**
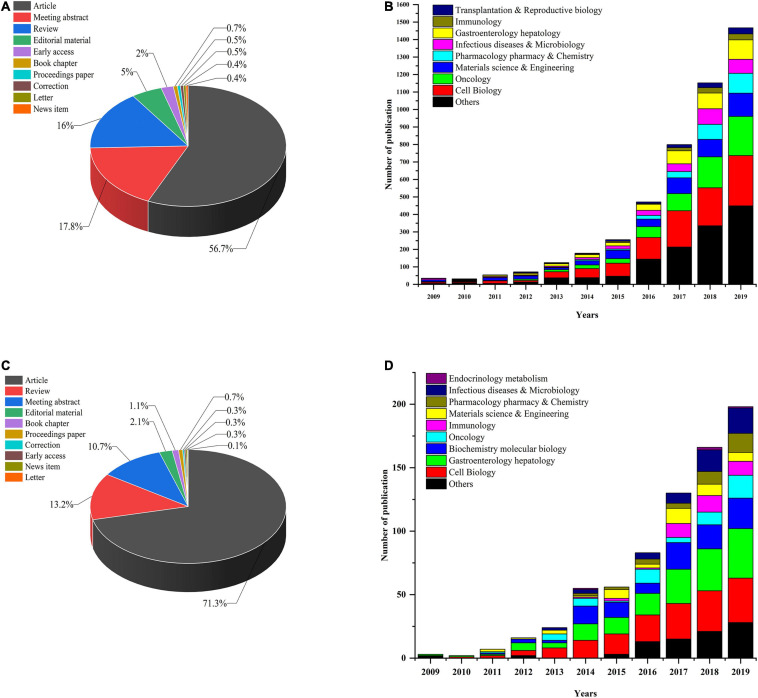
**(A)** Organoid research papers by document type percentage; **(B)** distribution of different research fields and number of papers for organoids per year in 2009–2019. **(C)** IO research papers by document type percentage; **(D)** distribution of different research fields and number of papers for IOs per year since 2009.

First, we performed a search query using “organoid” or “organoids” on the Web of Science Core Collection (WoSCC) database from 2009 (which was when the first report on organoids was published) to December 31, 2019, and 5,379 publications were found. Next, we conducted another search using the following terms: “intestinal organoids” or “mini-gut” or “enteroids” and identified 750 publications in all. These studies could be classified into 10 study types (see Figure 1C). Original articles (71.3%) were the most dominant type of publications throughout the whole period, and reviews accounted for 13.2% of articles. We used the polynomial model (see Figure 1D) to identify the number of publications on IO research in various research fields. The successful development of IOs was first reported in 2009, and the next 5 years showed slow and steady progress in the field. After 2015, the number of papers continued to show a growth trajectory, with a year-to-year increase in studies. Most research has mainly focused on cell biology and oncology in the fields of gastroenterology and hepatology. In addition, the number of papers published in immunology, pharmacology/pharmacy and chemistry, materials science and engineering, and infectious diseases and metabolism per year steadily increased. A similar trend of annual development was observed for both the studies on organoids and IOs (see Figure 1).

### Network Analysis of Research Topics

**FIGURE 2:**
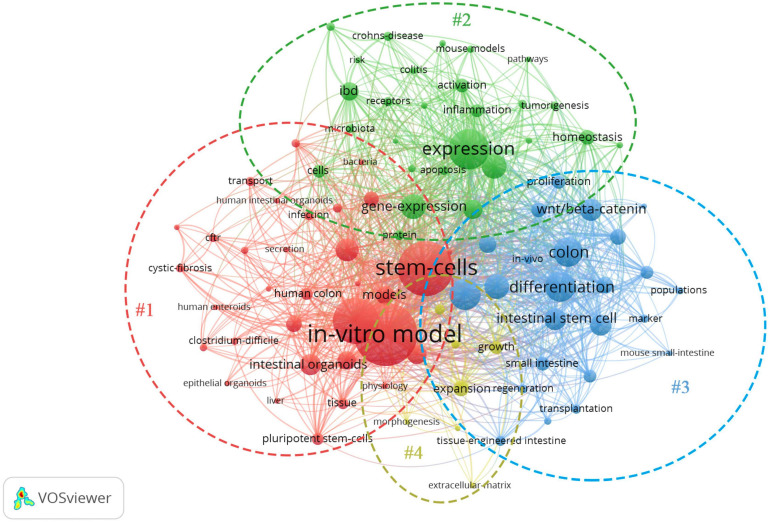
Network map of the high-frequency keywords for IO research.

The network map of keywords was visualized by VOSveiwer 1.6.11 (Leiden University, Leiden, Netherlands). The node size in the network map represents the frequency of keywords, the links between nodes represent their co-occurrence, and the differences in node color indicate different clusters (Xie, 2015; Gao et al., 2019). Network analysis based on high-frequency keywords (see Figure 2) showed that the keywords from published papers could be clustered into four groups. The red region (Cluster #1, entitled “The formation of IO”) included the main terms associated with stem cell and organoid formation, including stem cells, IOs, pluripotent stem cells, pathogenesis, human colon, enteroids, culture, and disease. The green region (Cluster #2, entitled “IO as a disease model”) described the application of organoids in various fields, such as disease models and drug screening. The terms in the figure (Crohn’s disease, tumorigenesis, homeostasis, colitis, microbiota, etc.) represent the current, relatively mature field of organoids. The blue area (Cluster #3, summarized as “IO for drug research”) indicated that tissue-engineered intestine, including tissue transplantation and regeneration, could represent a new era in IO applications. Another interesting finding from these network maps was the yellow cluster, which was related to engineered biomaterials used in organoid systems (Cluster #4, indicated by “IO in organ regeneration and transplantation”), representing potential ways for overcoming the shortcomings of organoid systems. Although there are few studies on this topic at present, we believe that this topic will become the next hot spot in organoid research.

**FIGURE 3:**
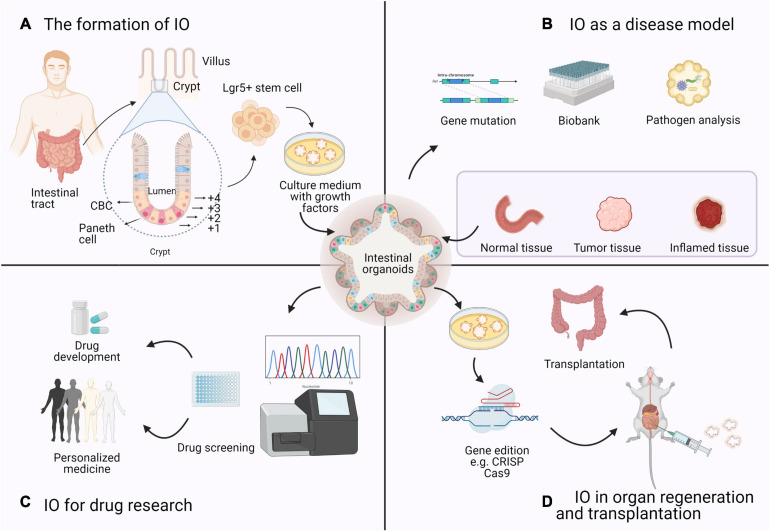
Four research topics on the application of IOs based on bibliometrics. **(A)** The formation of IO: Organoids are derived from a single Lgr5 + ISC. ISCs reside either at the crypt base, between Paneth cells, as CBCs, or at position +4 from the bottom of the crypt. **(B)** IO as a disease model: Intestinal diseased organoids use samples from biopsies or resection and preserved as a biobank in basic research. **(C)** IO for drug research: Based on the sequencing results and gene–drug association links, high-throughput drug screening of candidate drugs can be performed in IOs to determine effective therapeutic strategies. **(D)** IO in organ regeneration and transplantation: Many studies of organoid transplantation and maturation are in progress, which means that organoids can be used as a source of cells or tissues with genetic mutations for regenerative medicine purposes.

Based on the bibliometric findings, we will highlight recent advances and divide them into four research topics related to the application of IOs over the past decades with the expectation of more valuable conclusions in future follow-up studies (see Figure 3).

## Applications of IOs According to the Four Clusters of the Network Analysis

### The Formation of IOs

The gastrointestinal tract is an essential part of the human body responsible for digestion, nutrient absorption, and waste excretion (Okamoto and Watanabe, 2016). The intestinal epithelium is a single-cell layer that establishes a natural barrier against the external environment through a highly regulated self-renewal and differentiation process (Snijder and Pelkmans, 2011; Yui et al., 2012; Barker, 2014). Crypts are proliferative areas that harbor stem and progenitor cells at the base, and villi are differentiated regions that protrude into the lumen and consist of various terminally differentiated cell types; these two areas constitute the basic structure of self-renewing epithelial cells. Intestinal stem cells (ISCs) are indispensable at the start of the canonical crypt-to-villus hierarchical migratory hypothesis (Hendry and Potten, 1974; Bjerknes and Cheng, 2006). Two subgroups of ISCs can be identified: ISCs reside on the bottom of the crypt and are also referred to as crypt base columnar (CBC) cells, while +4 region cells stay in a quiescent state (Scoville et al., 2008). The intestinal epithelium maintains regenerative capacity and can be replaced every 3–4 days in mice and every week in humans. The specialized ISC population in the CBC is the main driving force of intestinal self-renewal (Sato et al., 2009; Kwon et al., 2020). For the sustainable maintenance of the intestinal epithelium, CBC cells that strongly express leucine-rich repeat-containing G protein-coupled receptor 5 (Lgr5) (Barker et al., 2007) provide progenitor cells during self-renewal (see Figure 3). Generally, Lgr5 + CBC cells differentiate into transit-amplifying (TA) cells, which migrate upward along the crypt–villus axis and further lose their proliferative ability, and finally generate mature epithelial cells, such as enterocytes, goblet cells, enteroendocrine cells, Paneth cells, and Tuft cells (Sato et al., 2011b; Yui et al., 2012).

Sato et al. (2009) utilized this self-organizing and self-renewal characteristic of ISCs to develop the first IO culture system from a single Lgr5 + ISC in mice. Accordingly, niche-mimicking and defined growth factors are added to the culture medium to either stimulate or inhibit signaling pathways involved in sustaining the self-renewal capabilities of the Lgr5 + ISCs (van der Flier and Clevers, 2009; Sato et al., 2011b; Clevers et al., 2014; van Rijn et al., 2016). The important ingredients of the small IO culture medium include R-spondin 1; Holmberg et al., 2017; Qi et al., 2017), epidermal growth factor (EGF), bone morphogenic protein (BMP) antagonist Noggin, and ROCK inhibitor Y-27632. R-spondin 1 is a Wnt agonist that induces marked crypt hyperplasia *in vivo* (Barker et al., 2007). An expansion in the number of crypts is induced by Noggin’s transgene expression (Haramis et al., 2004). The Rho kinase inhibitor Y-27632 can inhibit anoikis of ESCs (Zhang et al., 2019), whereas WNT3A supplementation is necessary for colonic organoid cultures (Sato et al., 2009, 2011a). To reduce the commercial burden of these defined factors and simplify the media formulation for organoid culture, Miyoshi and Stappenbeck (2013) inverted a novel conditioned medium (CM) from the supernatant of L-WRN cells, which are derived from mouse L cells and secrete Wnt3aA, R-spondin 3, and noggin. Compared with recombinant media, L-WRN CM is relatively inexpensive and provides complete high-titer proteins to activate Wnt signaling pathways.

Morphologically, IOs recapitulate intestinal epithelial structures *in vivo*: single Lgr5 + ISCs initially form villus-like spherical structures with a closed-loop hollow lumen, and then the cyst buds up, differentiates into a crypt-like structure, and finally forms a mature organoid structure; subsequently, ISCs stay at the bottom of the budding domains, while other differentiated intestinal epithelial cells (IECs) migrate to the central cyst (Sato et al., 2009; Date and Sato, 2015). Moreover, IOs can maintain stable genetic characteristics and biological behavior after repeated freezing and passaging and require passaging at 1:3–1:4 every 3 days (VanDussen et al., 2015).

### IOs as a Disease Model

Spence et al. (2011) generated an organoid system to mimic embryonic intestinal development. This system recapitulates the differentiation from human pluripotent stem cells (hPSCs) to polarized intestinal epithelium that is patterned into villus-like structures and crypt-like proliferative zones and contains all major epithelial cell types. This breakthrough demonstrates that human intestinal organoids (HIOs) can be used to study early events in intestinal disease development and identify unique disease preventive agents. Finkbeiner et al. (2015) demonstrated that HIOs closely resemble the fetal intestine and found that the ISC marker OLFM4 was expressed at extraordinarily low levels in the fetal intestine and HIOs but expressed at high levels in adult crypts, indicating that HIOs can be used to model fetal-to-adult gut maturation. A dysfunctional mutation in NEUROG3 induces congenital malabsorptive diarrhea because of the lack of enteroendocrine cells. Furthermore, knocking down the NEUROG3 transcript *via* short hairpin RNA in hPSC-derived organoids has been reported to result in a reduction in intestinal enteroendocrine cells, suggesting that NEUROG3 expression can directly affect the development of intestinal enteroendocrine cells (Spence et al., 2011).

Over past decades, IOs have been widely used to study the pathophysiology of various human diseases, including colorectal carcinoma (CRC), gastrointestinal inflammation (such as inflammatory bowel disease, IBD), infectious diseases (such as those related to *Helicobacter* and *Salmonella*), malignancy, and genetic diseases (such as cystic fibrosis, CF).

#### Intestinal Carcinoma

Colorectal carcinoma is a heterogeneous disease that consists of genomic and epigenetic alterations (IJspeert et al., 2015). Organoids cultured from human CRC cells are more accurate models of CRC that present stable passage, easy operation, and indefinite expansion features. Correspondingly, transcriptomic profiling has shown that these “patients in the lab” can carry over the complete genotypic and biological characteristics of the original tumor (van de Wetering et al., 2015; Vlachogiannis et al., 2018; Lau et al., 2020).

Methods have been developed to culture CRC organoids originating from both surgery specimens and endoscopic biopsy tissues. Multiple collections of patient-derived organoid (PDO) cultures have been established. van de Wetering et al. (2015) collected tissue from 22 consecutive primary CRC patients and 19 normal controls to establish the first living organoid biobank, with a 90% success rate. Compared with normal hIOs, over 94% of IOs derived from CRC patients showed aberrant activation of the Wnt signaling pathway caused by mutations (Cancer Genome Atlas Network, 2012); therefore, Wnt and R spondin withdrawal culture medium was used to selectively expand tumor organoids with high purity (van de Wetering et al., 2015). Intestinally, further testing showed that in addition to Wnt activators, an optimal oxygen concentration and a p38 inhibitor were essential for the long-term passage of CRC organoids (Fujii et al., 2016). With improved culture conditions, living organoid biobanks representing rare histological subtypes (e.g., mucinous adenocarcinoma and neuroendocrine tumor), premalignant subtypes (e.g., hyperplastic polyps, sessile serrated adenoma/polyps, and tubulovillous adenoma), and metastatic CRC have been successfully established (Fujii et al., 2016). Another study (Vlachogiannis et al., 2018) obtained 110 biopsy specimens from patients with advanced colorectal and gastroesophageal cancer tissues and constructed 71 PDOs. According to the comparison of the drug response (including that of target therapies and chemotherapies) observed in patients and the *ex vivo* response in the respective PDOs, these PDOs have higher specificity and more accurate predictions than other models, indicating that they have the potential to be implemented in personalized medicine programs.

Sixty percent of all CRC cases arise from adenomatous polyps following the adenoma-carcinoma sequence (also named the conventional pathway) (Lau et al., 2020; Shaashua et al., 2020). Wnt signaling is continuously activated in the intestinal organs of APC-deficient mice, which interferes with the differentiation process. Transplanting APC gene-deficient organs into immunodeficient mice can cause subcutaneous tumor formation and activate the Kras signaling pathway to promote tumorigenesis (Onuma et al., 2013). Cao et al. (2015) established IOs derived from CRC-prone Apc^Min/+^ mice to screen for epigenetically active compounds responsible for increasing organoid differentiation [including histone deacetylase (HDAC) inhibitors, sirtuin (SIRT) modulators, and methyltransferase inhibitors]. Xu et al. (2014) used Apc^Min/+^ mouse intestinal epithelium to form APC gene-deficient heterozygous organs to mimic familial adenomatous polyposis and found that Rad21 is a crucial regulator of APC gene deficiency and plays a key role in the pathogenesis of colon cancer. Nadauld et al. (2014) found that TGFBR2 loss induces metastatic gastric cancer *via* shRNA knockdown in Cdh1^–/–^ Tp53^–/–^ murine organoids. With similar shRNA strategies, the Apc, p53, Kras^G12D^, and Smad4 genes were knocked down in murine CRC organoids. These organoids exhibited an invasive adenocarcinoma-like histological structure and had tumorigenic tumors *in vivo* (Li et al., 2014). In 2018, scientists (Sakai et al., 2018) transplanted intestinal tumor-derived organoids carrying mutations in APC, Kras^G12D^, and TGFBR2 into mice, and these organoids promoted intravasation and efficient liver metastasis.

Efficient genome cleavage by the CRISPR-Cas9 system in organoids contributes to defined gene defects mimicking the effect of tumors. By knocking out tumor suppressor factors, such as APC, TP53, and SMAD4, as well as overexpressing oncogenes, such as Kras and PI3K, by CRISP/Cas9, direct conversion of healthy human colon organoids into homologous cancerous organoids has been realized (Drost et al., 2015; Matano et al., 2015). Deleting certain DNA repair genes to simulate mismatch repair deficiency in human CRC organoids and dissecting predominant mutation signatures could be performed to systematically determine their molecular origins (Drost et al., 2017). Serrated colon adenoma is a precursor CRC subtype that is characterized by a serrated histopathological morphology and initiated by oncogene BRAF^V600E^ mutations. Scientists have demonstrated that BRAF^V600E^ mutations in combination with microenvironmental transforming growth factor-β (TGFβ) signaling drive the transformation from serrated colon adenoma to mesenchymal CRC in engineered BRAF^V600E^-expressing human organoid cultures (Fessler et al., 2016). CRISPR-Cas9-based orthotopic transplantation models of organoids have also been established (O’Rourke et al., 2017; Roper et al., 2017). Organoids harboring Apc/Trp53 deletion were directly injected into the colonic epithelium for primary tumor and hepatic metastasis formation in mice without cancer-predisposing mutations. To promote high-throughput genetic testing and the functional characterization of tumor drivers, the latest studies developed a screening platform that uses a combined CRISPR-Cas9 library in PDOs from CRC patients, and it can identify tumor suppressor genes *in vivo* and *in vitro*. The platform is paired with a unique molecular identifier (UMI) to establish a CRISPR-UMI screening library for patient-specific functional genomics, thus allowing phenotypic research at a clonal level (Michels et al., 2020). Accordingly, organoids derived from a single cell that capture the intratumor heterogeneity of genetic mutations can be a powerful tool to improve therapeutic strategies against CRC. Notably, in the PDO system, multiple passages should be avoided to ensure that the genetic features of the original tumors are maintained and prevent the continuous accrual of chromosome mis-segregations or replication errors in organoids with chromosomal instability.

#### Inflammatory Bowel Disease

Inflammatory bowel disease can cause damage to the epithelium of the small intestine (Crohn’s disease, CD) and colon (ulcerative colitis, UC). The lack of an appropriate model for the intestinal epithelium in IBD has hindered studies on the possible mechanisms and drug development. Dotti et al. (2017) established UC epithelial organoids first and identified differential gene sets between UC and normal organoids. Interestingly, the genes upregulated in UC, including WNT3, EGF, DLL4, and BMP2, are key niche factors of Paneth cells in the murine small intestine. Another study used PDOs taken from the active lesions of CD patients and examined them *via* microfluid-based single-cell multiplex gene expression analysis (Suzuki et al., 2018). The results showed that the active lesion presents a distinct expression pattern of ISC marker genes, suggesting that small ISC properties are modified by unidentified factors in the inflammatory environment. These studies indicated that the inflammatory microenvironment in the ISC niche leads to a disrupted epithelial barrier and suggested that impaired epithelial regeneration could be a potential cause of the pathogenesis of UC.

Previous studies have re-established the inflammatory milieu in IO culture systems. Mutation of the autophagy-related gene ATG16L1 can cause necrotic apoptosis of the intestinal epithelium and Paneth cell dysfunction related to IBD. Stimulation with tumor necrosis factor-α (TNF-α) or its combination with interferon-γ induced Paneth cell loss and programmed necrosis in ATG16L1-deficient IOs. The cytoprotective function of ATG16L1 is related to the role of autophagy in promoting mitochondrial homeostasis, indicating the role of ATG16L1 in protecting IECs (Matsuzawa-Ishimoto et al., 2017; Pott et al., 2018). ATG16L1 deficiency also sensitizes mouse ileal organoids to the cytokine interleukin (IL)-22, a Crohn’s disease-related cytokine that is associated with epithelial proliferation, tissue regeneration, inflammatory response, and transcriptional program regulation (Tsai et al., 2017; Powell et al., 2020). Studies have used the organoid survival rate as a determinant for ISC survival and organoid size as a measure of ISC proliferation, and they demonstrated that increased IL22 expression limits ISC expansion by controlling progenitor cell numbers and expansion (Zwarycz et al., 2019). Powell et al. (2020) analyzed transcriptomic data of PDOs from a large cohort of patients with active CD and found that IL22-responsive transcripts are highly correlated with the endoplasmic reticulum (ER) stress response transcription module of the colonic epithelium in active colitis patients. Both IL22 and ER stress are highly enriched in the colonic epithelium of active colitis patients and are positively correlated with the severity of colitis, suggesting that the IL22/ER stress axis plays an essential role in chronic inflammation development. *Via* whole-exome sequencing of uninflamed and inflamed organoids from the same UC patient, a recent study demonstrated that the inflamed epithelium of UC accumulates somatic mutations related to the IL-17–NF-κB signaling pathway. Notably, some of these mutations could exacerbate IBD but are irrelevant to tumorigenesis (Nanki et al., 2020). Khaloian et al. (2020) found that ileal crypts from inflamed TNF^ΔARE^ mice with mitochondrial dysfunction caused by knocking out Hsp60 in Lgr5 + ISCs failed to grow mature organoids. Mitochondrial respiratory dysfunction resulted in lower Lgr5 expression in ISCs and promoted their differentiation into abnormal Paneth cells to influence the ISC niche. Taken together, these findings indicate that the IO model can be effectively used to provide insights into the mechanism of IBD according to pathways specific to the epithelium and genotype, and that it can also be used to identify new therapeutic targets.

#### Cystic Fibrosis

Intestinal organoid technology has had impressive impacts on research for the autosomal recessive disorder, CF, which is caused by mutations in the CF transmembrane conductance regulator (CFTR) gene that contribute to aberrant function of the chloride channel (Ratjen and Doring, 2003). Patients with CF suffer from the buildup of highly viscous mucus, and the main complications are persistent lung and gastrointestinal tract infections (Dekkers et al., 2016). Because various CFTR mutations (up to 2,000) are implicated in a broad spectrum of phenotypes, cell lines and animal models cannot faithfully mimic the self-renewal of CF-related cells (Dekkers et al., 2013). Because of the absence of well-defined molecular targets, CF patients lack effective treatment options.

The earliest use of IOs in CF was reported in 2012 (Liu et al., 2012): researchers used murine intestinal crypt cultures for physiological studies of crypt epithelium by focusing on the transport activity of the CFTR. Subsequently, Dekkers et al. (2013) collected ISCs from rectal biopsy specimens to generate organoids and developed a sophisticated microscopic assay called the forskolin-induced swelling (FIS) assay to model CFTR function. CFTR is the only channel that opens in a cAMP-dependent manner in the intestine. Forskolin increases the intracellular cyclic AMP concentration, thereby inducing the robust swelling of wild-type organoids. Correspondingly, swelling does not occur in CF organoids with abnormally functioning CFTR, is attenuated in organoids expressing CFTR-F508del (F508del is the most dominant CFTR mutation), and is totally lost in CFTR-deleted organoids. By applying PDO technology and FIS assays, this team demonstrated that cotreatment with the CFTR potentiator VX-770 and the CFTR corrector VX-809 was superior to a single treatment in restoring the function of CFTR-F508del (Dekkers et al., 2016). In addition, Clevers’ team (Schwank et al., 2013) modified the CFTR locus by homologous recombination *via* CRISPR/Cas9 technology, wherein a normal CFTR gene was inserted into organoids from CF patients. The morphology, function, and gene expression of these PDOs were consistent with those of healthy organoids, and FIS was restored after repair. If these repaired organoids are reintroduced into the source patient, they may cure CF. Thus, organoid technology provides a new scope for personalized treatment for CF patients.

#### Infectious Diseases

The human gastrointestinal epithelium is the prime interface for interactions with microorganisms. The healthy intestinal mucosal barrier is a finely tuned ecosystem of microbiota that maintains the balance between host–microbe interactions. Intestinal cell lines and animal models cannot accurately imitate gastrointestinal infectious diseases *in vitro* because of poor phenotypic, genetic, and epigenetic characteristics (Yin et al., 2015). Increasing studies are focusing on explaining the relationship between host–pathogen interactions in IO models. 3D enteric pathogen organoid models, including bacteria, viruses, and parasites, have been successfully established to explore the physiological role or pathogenic mechanism of the intestinal infectious diseases. Instances of such infectious agents include *Salmonella typhi* (Zhang et al., 2014; Wilson et al., 2015), enterohemorrhagic *Escherichia coli* (EHEC) (Foulke-Abel et al., 2014; In et al., 2016), *Clostridium difficile* (Engevik et al., 2015; Leslie et al., 2015), rotavirus (Saxena et al., 2016), norovirus (Ettayebi et al., 2016), enteroviruses (Drummond et al., 2017), and *Cryptosporidium* (Heo et al., 2018).

Many of the pathogens that infect humans fail to grow in 2D cultures. Although several attempts have been made over many years, scientists are still unsure how to grow human norovirus (HuNov) *ex vivo* because of the lack of a reproducible cultivation system. Research has used microinjections to mimic enteric infections, and this approach is more beneficial for the intestinal lumenal structure (Engevik et al., 2015). Ettayebi et al. (2016) established an ISC-derived organoid system to support HuNov replication *in vitro* and demonstrated that some variants (such as GII.3) replicate only in the environment of the intestinal cellular milieu constructed by bile, suggesting that IOs might be used as antivirals against norovirus. Rotaviruses are a common cause of acute, dehydrating, often fatal, gastroenteritis in infants and young children. Finkbeiner et al. (2012) tested that induced hIOs derived from differentiated stem cell lines can support replication of rotaviruses directly from stool samples. Compared with traditional cell line models, most rotavirus strains have about 10 times higher viral replication power, which supports the use of HIO as a new tool for human rotavirus research. Meanwhile, Heo et al. (2018) infected intestinal and lung organoids derived from healthy human donors with *Cryptosporidium parvum.* Of note, *Cryptosporidium* propagates within IOs, especially in differentiated organoids rather than expanding organoids, and recapitulates its complex life cycle with asexual and sexual stages *in vitro*. Present studies confirm that IO can also be a promising model for vaccines and antivirals. Yin et al. (2015) demonstrated that compared with 2D Caco-2 (immortalized human colonic adenocarcinoma) cell lines, hIOs are highly permissive to rotavirus infection. Furthermore, treatment of interferon-alpha or ribavirin that inhibited viral replication in organoids showed more sensitive and diverse antiviral effects. Next, this team testified the effects of PI3K–Akt–mTOR signaling on maintenance of rotavirus infection within hIOs, and the mTOR inhibitor, rapamycin, induced autophagy machinery to inhibit rotavirus replication, which may indicate that new therapeutic targets for antiviral drugs.

The threat caused by severe acute respiratory syndrome coronavirus 2 (SARS-CoV-2) is challenging health systems globally. Regarding the ongoing coronavirus disease 19 (COVID-19) pandemic, human organoids are also being utilized against SARS-CoV-2 infections (Hou et al., 2020; Wang et al., 2020; Zhou et al., 2020; Geurts et al., 2021). Compared with many cell lines, human organoids can be infected with SARS-CoV-2 easily without inducing the addition of key host factors, such as ACE2, which allows the examination of pathogen interactions with primary epithelial cells directly. These unanticipated findings provide new insight into enteroviral infection using IOs that provide a robust model system for studying rotavirus–host interactions and assessing antiviral medications.

### IOs for Drug Research

The intestinal epithelium contains several drug-metabolism enzymes that play a vital role in the assessment of the pharmacokinetics of oral drugs, the metabolism of drugs, and the expression of uptake and efflux transporters (Onozato et al., 2018). The development of novel drugs is difficult, and the translation of innovative science into effective therapies has been seen as a major bottleneck in new drug research. Compared with the traditional drug screening model, IOs predict drug toxicity and human efficacy more accurately, thus making them an important alternative in drug screening and development that can provide perspective and highlight emerging opportunities.

Regarding pharmacokinetic functions, IOs generated from murine intestinal cells were successfully used to evaluate the function of the efflux transporter ATP-binding cassette, subfamily B, member 1/multidrug resistance 1 (ABCB1/MDR1) (Hilgendorf et al., 2007; International Transporter Consortium et al., 2010). In recent years, studies quantifying the excretion function of ABCB1/MDR1 using 3D IOs have been reported, and these organoids are expected to be applied to a P-gp inhibitor screening system (Mizutani et al., 2012; Zhang et al., 2016; Zhao et al., 2017). Onozato et al. (2018) successfully induced human iPSC differentiation in pharmacokinetically functional IOs by using A-83-01, PD98059, 5-azacytidine, and dual antiplatelet therapy (A/PD/5-aza/DAPT). This result indicates that human iPSC-derived IOs can serve as useful *in vitro* experimental systems in pharmacokinetic studies and accelerate the pace of mechanistic research.

Grabinger et al. (2014) found that IOs were 10–30 times more sensitive to 5-fluorouracil treatment than 2D cell lines *via* a modified MTT assay, suggesting that *ex vivo* IOs may reflect the sensitivity of intestinal crypt cells more closely *in vivo*. Next, they cultured IOs from mice with deletion of the pro-apoptotic Bcl-2 homolog Bim (Bim^–/–^ mice) and wild-type mice and treated them with cisplatin, revealing that the sensitivity of IOs from Bim^–/–^ knockout mice to cisplatin was significantly decreased and indicating that Bim is necessary for IO apoptosis induced by cisplatin. Another study (Lavitrano et al., 2020) used drug-resistant TP53-null colon cancer PDOs and demonstrated that silencing or chemical inhibition of p65BTK, a novel oncogenic isoform of Bruton’s tyrosine kinase (BTK), helped overcome the 5-fluorouracil resistance of PDOs and significantly slowed the growth of xenografted tumors. Targeting p65BTK can restore the apoptotic response in drug-resistant CRC cells, suggesting that the combination of BTK inhibitors with 5-fluorouracil is a novel therapeutic approach for CRC patients.

High-throughput screening of 83 types of compounds and 22 types of living organoids from biobanks was applied to identify the most potent drugs for each organoid, and then the drug sensitivity was correlated with the genomic data to accurately detect the molecular markers related to drug effects and lesions. This organoid drug screening assay generates reproducible high-quality drug-sensitivity data. Vlachogiannis et al. (2018) collected 67 metastatic CRC biopsy samples from 61 patients and cultured 40 PDOs. These PDOs could be used to predict the patient response to irinotecan (I) and I + 5-fluorouracil (FI) combination therapy, which demonstrated that a response to FI combination therapy in a PDO indicated longer progression-free survival in the corresponding patient. This report was the first to show the prognostic value of PDOs in personalized medicine *via* a biobank of PDOs. Moreover, Buzzelli et al. (2018) validated that CRC liver metastasis organoids recapitulated the morphological characteristics and pathological stage of the corresponding tumor. These organoids were collected from patients who did not respond to oxaliplatin treatment or capecitabine therapy. The cultures were pretreated with three rounds of chemotherapy drugs for 4 days, and the loss of distinct lumen-like structures in the culture morphology was observed after chemotherapy. The results demonstrated that CRC liver metastasis organoids acquire chemotherapy resistance patterns and can be used as surrogates for drug testing. Furthermore, an additional study (Toden et al., 2018) using PDOs demonstrated that oligomeric proanthocyanidins (OPCs) in CRC protect against chemotherapy-induced toxicity by targeting cancer stem cells. Another study involving an organoid culture assay revealed that apigenin (Xu et al., 2016) suppresses CRC cell proliferation, migration, and invasion *via* inhibition of the Wnt/β-catenin signaling pathway. The results discussed above indicate that IOs can recapitulate the histological and genetic features of CRC tissues and may serve as useful drug screening models.

Organoids help bridge the gap between preclinical and clinical research by providing a relevant *in vitro* model of human diseases. The combination of newly established 3D patient organoid culture systems with personalized high-throughput drug screening and genomic analysis of patient-derived tumor samples offers a unique opportunity to stratify and identify effective cancer therapies for individual patients (Kriston-Vizi and Flotow, 2017).

### IOs in Organ Regeneration and Transplantation

Organ transplantation is currently the best method for treating end-stage organ diseases. However, multiple problems, such as organ source, postoperative graft rejection, and complications, remain to be solved. Proof-of-concept studies have demonstrated the feasibility of expanding organoids from single stem cells and subsequent safe transplantation into animals.

Transplanting functional colonic organoids into a dextran sulfate-induced acute colitis mouse model can repair the damaged colonic epithelium, suggesting that organoids can expand from single adult colonic stem cells *in vitro* (Yui et al., 2012). Sugimoto et al. (2018) transplanted human colon organoids *in situ* into the submucosa of mouse colons and observed their growth in mice. Interestingly, the xenografts retained the original human characteristics rather than acquiring those of the mouse host; these features included the crypt–villus structure and Paneth cells. Patients with IBD who fail to respond to physical medication have to undergo enterectomy, which leads to a substantial reduction in quality of life. Yui et al. (2012) grafted mouse-derived colon organoids to cover the epithelial-defective region in mice with colitis. Four weeks later, the organoid-based xenografts had formed a single-layered epithelium that restored normal intestinal function and histology. A clinical trial (Shimizu et al., 2019) established an orthotopic xenograft system for IBD patients *via* organoids derived from colonoscopy biopsy samples. In the trial, the generated grafts enriched in ISCs were used to cover the wound, thus enabling the restoration of mucosal barrier integrity and local immune abnormalities. Therefore, ISC transplantation represents a potential therapeutic tool for IBD patients who do not respond to medication and refuse surgery. Nakamura and Sato (2018) proposed the use of tubular scaffolds to culture IOs into a tubular structure *in vitro* and showed subsequent anastomosis to the intestinal defect site *in vivo* after induction of differentiation. Furthermore, a panel of immunosuppressants was screened using human mini-guts to confirm that mycophenolic acid potently inhibits rotavirus infection by inhibiting inosine-50-monophosphate dehydrogenase (IMPDH) in the host, which indicates the possible dual benefits of mycophenolic acid in preventing organ rejection in transplantation patients and combatting rotavirus infection (Yin et al., 2016). In addition, various reports have described the development of transplantation procedures for gene-edited organoids that enable the generation of metastatic mouse models of CRC (see section “Intestinal Carcinoma”). These methods reveal the potential therapeutic applications of IOs in regenerative medicine; however, they are only theoretically feasible and need to be supported by clinical trial results.

Nonetheless, successful *in vivo* organoid-based engraftment of epithelial cultures remains challenging because of the lack of a supporting mesenchyme. Researchers in Ohio transplanted ESC-derived or iPSC-derived IOs into immunodeficient mice, and these HIOs not only formed mature human intestinal epithelium but also generated a laminated human mesenchyme (Watson et al., 2014). This research paved the way for future bioengineering studies. Recently, scientists (Gjorevski et al., 2016; Cruz-Acuna et al., 2017) performed decellularization, lyophilization, and grinding; radiation sterilization; digestion and neutralization; and other treatments on porcine small intestinal mucosa to produce extracellular matrix (ECM), which can retain nutrients such as collagen, elastin, and mucopolysaccharides. The mechanical properties and 3D structure were comparable to those of commercial Matrigel, and it has been verified that the characteristics of the ECM proteome are similar to those of human endoderm stem cell-differentiated tissues, which can successfully cultivate human and mouse endoderm-like organoids (such as stomach, liver, pancreas, and small intestine). In addition to the ECM from animal tissue, the growing demand for non-xenobiotic materials is desirable for the application of human organoids. Gjorevski et al. (2016) established a new protocol by facilitating the required minimal adhesion by adding proteins such as RGD and laminin-111 to a poly ethylene glycol (PEG) hydrogel backbone. Poling et al. (2018) confirmed the importance of dynamic mechanical forces in HIO morphogenesis. A nitinol spring covered by a degradable capsule was implanted inside the HIOs, and it resulted in a significant increase in villus height, crypt depth, and crypt fission compared with that of the control HIOs. In addition to the similarity of the combination of the spring and HIOs to the human jejunum in morphology, the ISC compartment, vilification process, and smooth muscle thickness in the organoids plus spring group were significantly increased, indicating better barrier functions and smooth muscle motility. This new method is expected to accelerate the application of intestinal transplantation technology. These developments in biomaterials reveal the potential for the clinical application of human organoids.

In summary, IOs have been established from a range of different diseases, which lays a foundation for more studies on drug development and precision medicine. Of note, while autologous cell therapy transplantation is highly promising in the organoid field, its efficacy, safety, and immunogenicity are still pending evaluation.

## Limitations and Development

Compared with traditional models, IO technology opens new horizons for disease research in areas including organogenesis, cellular differentiation, genomic analysis, cell–cell interaction, and physiological functions (see Table 1). This novel research tool has sufficient data support for large-scale experimental research. However, several shortcomings remain. First, an important inherent limitation of organoid culture is the lack of a mesenchymal structure, vasculature factors, and immune cells; accordingly, co-culture systems should be explored in the context of organoids. Second, although Matrigel-based IO culture systems can largely simulate the human intestinal epithelium, their spherical structure prevents external stimuli from entering the apical region, thus limiting their high-throughput testing, drug interactions, and microbial–epithelial interactions (Altay et al., 2019). Third, the enclosed lumen structure limits the secretion of materials, leads to the shedding of apoptotic cells from the luminal region, and influences the efficacy of drugs.

New insights have been reported regarding the abovementioned concerns. Ettayebi et al. (2016) first established human intestinal enteroid monolayer cultures that were inoculated with HuNov. In addition, a method of culturing organoids in a “hanging drop” without embedding them in Matrigel has attracted great interest, and this technique physically enhances cell-to-cell interactions due to the lack of rigid support (a glass or plastic surface) or a solidified ECM scaffold (Foty, 2011). Ueda et al. (2010) used mouse iPSCs to organize an enteric organoid with motor functions *in vitro* by a hanging drop culture system, which exhibited spontaneous contraction and highly coordinated peristalsis accompanied by the transportation of contents. Polish scientists (Panek et al., 2018) also formed organoids derived from chicken embryo intestines by applying a hanging drop system without embedding; this culture technique presents cost savings because it uses a smaller quantity of culture media and Matrigel. In addition, the solidification step of Matrigel is skipped, increasing the speed of the cell seeding process.

To overcome the obstacles in mimicking *in vivo* physiological coupling, a microfluidic-based 3D system called organ-on-a-chip has been developed (Bhatia and Ingber, 2014; Bein et al., 2018; Mittal et al., 2019). Organ-on-a-chip allows for two parallel hollow microchannels to be separated by a porous ECM-coated membrane. Kasendra et al. (2018) established a chip wherein human enteroids were cultured on an ECM-coated (Matrigel-type I collagen) membrane, and differential cells exposed their apical surfaces to an open lumen and interface with endothelium. In addition, this chip was reported to replicate normal intestinal functions, including nutrient absorption and mucus secretion. Recently, Liu et al. (2015) successfully mimicked the tumor microenvironment (TME) by co-culturing cancer cells and four TME-related cell types (fibroblasts, macrophages, human umbilical vein endothelial cells, and stromal cells) in a microfluidic device. The TME characteristics of paracrine interactions and macrophage migration were simulated in this system to enable the prediction of the effects of neoadjuvant chemotherapy. Taken together, these findings indicate that this novel technology can help better improve the efficacy and function of IOs.

Despite the remaining challenges, the future application of hIOs is still promising. The feasibility of using IOs as an accurate and high-throughput preclinical tool for precision medicine is being confirmed continuously. Given their comparable structure and behavior, IOs may serve as a relevant surrogate system for *in vitro* testing of intestinal epithelium-damaging drugs and toxins and for investigating cell death pathways, which will provide information on treatments that are ineffective for the patient, thus conserving valuable time for the development of effective therapeutic approaches for specific patients. The efficient culture of organoids makes it possible to screen individualized drugs within a clinical treatment time window and then to apply them to translational medicine and individualized treatment. It is believed that with an increased understanding and further innovations in organoid-related research, organoid culture will play an increasingly important role in scientific research.

The long-term IO model from single stem cells was first developed in 2009. In recent years, a growing body of literature has demonstrated that organoid technology has expanded to embrace genetic editing, omics-based drug screening analyses, and diverse co-culture systems with immune cells, viruses, bacteria, and parasites. Although IO culture protocols *in vitro* are relatively mature, the most urgent problems to be solved are how to characterize and verify that the established organoids present high fidelity with human biology and how to translate them from human organoids *in vitro* to regenerative transplantation approaches *in vivo*. Deep profiling of the organoids using spatially resolved single-cell RNA sequencing could be a powerful tool for revealing the subgroups, differentiation degree, and state of cells. The “Organoid Cell Atlas” pilot project is currently ongoing to address the problems mentioned above (Bock et al., 2021). We believe that human organoid systems will provide unprecedented opportunities to improve human health.

## Author Contributions

M-MZ, Y-CC, SW, and K-WJ designed the review. K-LY and QW performed the literature search and analyzed the bibliometric data. Y-SZ and H-RZ assisted in publication searching and screening. M-MZ and K-LY wrote the manuscript. Y-CC, Y-JY, SW, and K-WJ reviewed and edited the manuscript. All authors contributed to the article and approved the submitted version.

## Conflict of Interest

The authors declare that the research was conducted in the absence of any commercial or financial relationships that could be construed as a potential conflict of interest.

## Publisher’s Note

All claims expressed in this article are solely those of the authors and do not necessarily represent those of their affiliated organizations, or those of the publisher, the editors and the reviewers. Any product that may be evaluated in this article, or claim that may be made by its manufacturer, is not guaranteed or endorsed by the publisher.
